# Outdoor ground surface influences spatiotemporal parameters of walking for individuals undergoing rehabilitation: An observational study

**DOI:** 10.1371/journal.pone.0337177

**Published:** 2025-12-02

**Authors:** Benjamin S. Killen, Stacy Harris, Alan Levinson, Catherine Hamilton, Michael D. Lewek

**Affiliations:** 1 Human Movement Sciences Curriculum, University of North Carolina at Chapel Hill, Chapel Hill, North Carolina, United States of America; 2 Division of Physical Therapy, Department of Health Sciences, University of North Carolina at Chapel Hill, Chapel Hill, NC, United States of America; 3 Center for Rehabilitation Care, University of North Carolina Health, Chapel Hill, North Carolina, United States of America; University of Illinois Urbana-Champaign, UNITED STATES OF AMERICA

## Abstract

Clinical measures of walking capacity may differ from walking performed in outdoor environmental settings. This study assessed spatiotemporal aspects of gait in individuals undergoing rehabilitation when walking over various outdoor surfaces and compared them to a standard clinical gait measure. We sought to identify how spatiotemporal aspects of gait change across terrains commonly encountered in the community in individuals undergoing rehabilitation. Forty-six individuals with mobility deficits undergoing outpatient physical therapy completed the 10-meter walk test in clinic and walked over six standardized terrains (e.g., pavers, gravel, sand). We calculated gait speed, cadence, average step length, and walk ratio (calculated as cm/steps/min) from video recordings for all conditions and used repeated measures ANOVAs to assess differences between terrains compared to the 10-meter walk test. We then used multiple linear regressions to examine spatiotemporal mechanisms underlying gait speed changes across terrains. Gait speed and cadence were reduced when participants walked over sand, mulch, up sloping gravel, and down sloping gravel compared to the 10-meter walk test. Step length decreased in the sand, gravel upslope, and gravel downslope conditions compared to the 10-meter walk test. Walk ratio was decreased in the sloped gravel conditions. Cadence and step length changes explained 95–99% of the variance in gait speed changes across conditions with step length being more heavily weighted across conditions. Individuals undergoing physical therapy for mobility deficits walked slower, with smaller step lengths and cadence when faced with uneven outdoor terrain compared to an indoor 10-meter walk test while walk ratio remained consistent across level surfaces. Speed reductions were related to both step length and cadence. Walk ratio remained consistent across level terrains, indicating a consistent central control strategy, despite terrain changes. Although standardized measures like the 10-meter walk test provide critical information about gait capacity, they may not fully reflect performance in all real-world environments.

## Introduction

Physical rehabilitation often focuses on restoring the ability to independently move within one’s community environment (i.e., functional mobility). To assess mobility, physical therapists frequently measure movement domains like gait speed using standardized, clinical outcomes such as the 10-meter walk test (10MWT). Such measures of walking capacity are important because they are associated with functional mobility status and falls risk [1]. Decreased gait speed, for example, is a consequence of many injuries and disorders [2–5], and can lead to reduced participation in community activities and increased safety concerns, particularly in urban areas where gait speed demands may be increased [6,7].

To interpret findings in those with deficits in functional mobility [2–5], normative values for gait speed have been established using the 10MWT for adults at varying levels of community ambulation [8–10]. Importantly, however, these normative and threshold values were derived from tests conducted in well-lit, indoor clinical settings on unobstructed, flat surfaces which may limit the generalizability of these values. In the community, individuals frequently contend with various slopes and surface textures, challenging balance and dynamic stability [11]. Because individuals undergoing physical therapy for functional mobility concerns often present with concomitant balance deficits, it is likely that walking over such terrains would require adjustments in gait to handle the increased balance challenge or risk of trip-related falls. Specifically, individuals may adjust step length and cadence [12,13], both of which can change with age [12], walking surface [11], and pathology [14]. Gait speed and its underlying spatiotemporal measures have been well characterized on smooth level indoor and outdoor surfaces [8,15,16], but few studies have addressed ambulation over varying terrains typically encountered in the community. Previous work comparing indoor and outdoor walking has primarily recruited a specific population [17,18], or has included only a few commonly encountered terrains like sidewalks or grass [16,17]. However, it remains unknown how indoor clinical measures of gait (10MWT) compare to a variety of typically encountered outdoor, community-level terrains in individuals with mobility deficits.

Beyond understanding the differences across terrains for spatiotemporal gait variables, it is important to understand how individuals with mobility deficits alter their gait on different terrains. Previous literature has demonstrated that comfortable gait speeds are dictated by a relatively constant step length-cadence relationship, known as the walk ratio (WR), in both younger and older adults without pathology [19]. Although the WR typically remains constant when changing speeds and walking over uneven surfaces, it may vary with more cognitively challenging activities like dual tasking, and lower walk ratios may be predictive of falls risk in older adults [20,21]. The WR has been explored on uneven surfaces in healthy older and younger adults, yet we are unaware of literature that examined how this relationship changes on uneven, outdoor terrains typically experienced in the community for individuals with pathological gait. From an intervention standpoint, it is prudent to understand which spatiotemporal characteristics of gait change in such environments, as well as how they change across terrains.

The purpose of this study was therefore to quantify the changes in gait speed and spatiotemporal parameters that occur between a smooth, firm clinic setting and various outdoor community-level terrains such as pavers, sand, up and down sloping gravel, and mulch. Second, we sought to describe how spatiotemporal aspects of gait (i.e., cadence and step length) influence these anticipated changes in gait speed. We hypothesized that gait speed would decrease when walking over outdoor, uneven terrains compared to the 10MWT due to reductions in both step length and cadence that may be necessary to maintain stability. We also hypothesized that changes in step length would contribute to greater changes in gait speed on uneven terrains compared to changes in cadence, given that individuals with impaired balance and gait tend to demonstrate decreased step lengths compared to those without mobility impairment.

## Materials and methods

### Study protocol

Using an observational cross-sectional design, we examined gait changes in individuals undergoing physical therapy to improve functional mobility, while completing an indoor 10MWT and walking over a variety of outdoor surfaces. Participants were recruited as a convenience sample from the Center for Rehabilitation Care at the University of North Carolina at Chapel Hill. Inclusion criteria required that participants were adults, participating in physical therapy services due to difficulty with walking and were able to walk with no more than contact guard assistance. Two Spanish-speaking participants participated with the assistance of an interpreter. All participants provided written informed consent by signing a consent form approved by the Institutional Review Board at the University of North Carolina at Chapel Hill (Approval # 17–1985). Participants were recruited between August 23, 2017, and November 30, 2017.

A total of 50 (28 female, 22 male) participants were recruited across multiple clinical diagnoses. One participant did not complete all walking conditions, and three participants did not follow instructions, excluding them from the study. This left a sample size of 46 (24 female, 22 male) participants for analysis (see Table 1 for participant demographics and description of outdoor conditions). Participant diagnoses included Parkinson disease (n = 11); stroke (n = 8); musculoskeletal complaints such arthritis, amputation, joint pain (n = 17); trauma events such as traumatic brain injury, spinal cord injury, pain due to motor vehicle crash (n = 4); and other conditions such as spina bifida, cancer-related pain, falls prevention, multiple sclerosis, shortness of breath with weakness (n = 7).

**Table 1:** Participant Demographic Information.

Sex	24 F, 22M
**Age, mean (SD)**	64.3 (16.1)
**Height in meters, mean (SD)**	1.7 (0.09)
**Weight in kilograms, mean (SD)**	76.9 (17.8)
**Body Mass Index in kilograms/meter** ^**2**^ **, mean (SD)**	27.0 (5.93)
**Falls History, N (%)**	
0	25 (54.3%)
1-2	15 (32.6%)
3-4	4 (8.7%)
5+	2 (4.4%)
**Baseline Assistive Device Use, N (%)**	29 (63.04%)
**Assistive Device Used during Test, N (%)**	10 (21.7%)
**Orthotic Use, N (%)**	9 (19.6%)
**Involved Side, N (%)**	
Left	15 (32.6%)
Right	12 (26.1%)
Bilateral	3 (6.5%)
Neither	16 (34.8%)
**Diagnosis, N**	
MSK	17
PD	11
CVA	8
Other	6
Trauma	4
**Temperature During Testing in Celsius, mean (SD)**	18.4 (11.1)
**Weather, N sessions**	
Cloudy	8
Partly Cloudy	2
Sunny	35
Not Reported	1

Summary of Participant Demographic/ Session information; CVA (Cerebrovascular Accident), F (female), M (Male), MSK (Musculoskeletal Diagnosis), n (number), PD (Parkinson Disease), SD (Standard Deviation)

Participants were evaluated during a scheduled physical therapy session in which they performed both the 10MWT and an outdoor walk over an outdoor course that included various terrains (described below). For the 10MWT, participants walked over a 10-meter indoor walkway on a linoleum floor, in a well-lit corridor free of obstacles. Previous literature indicates that at least two steps are needed to achieve steady state speed on level and sloped terrains [22]. Therefore, participants were provided with space in front of and beyond the walkway to account for acceleration and deceleration. Researchers instructed participants to “walk to the end of the walkway at a pace that is comfortable and safe for you.” The 10MWT is both reliable [3,8,23–25] and valid [24–26] across populations to characterize gait speed and is considered a core outcome measure in neurologic populations [27]. In most cases, the 10MWT was conducted prior to outdoor conditions, with a few exceptions due to time constraints surrounding the participant’s appointment time. Cadence and average step length were calculated from video recordings of the 10MWT.

**Fig 1:**
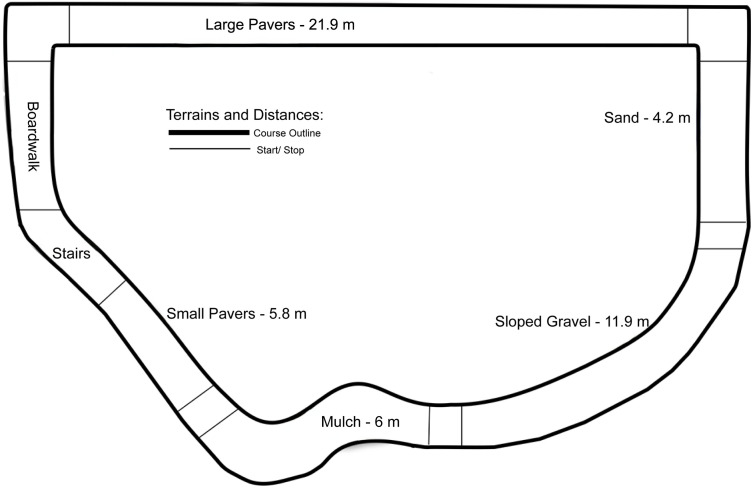
Diagram of Outdoor Terrains; Participants performed two loops (clockwise and counterclockwise). Start and Finish Markers are shown as well as distances for each condition. The boardwalk and stair conditions were not timed, but participants completed the entirety of the course.

Participants were video recorded using a handheld digital video camera (Sony HDR-CX150, Sony Corporation, New York, NY) while walking over an outdoor “challenge course” during their rehabilitation session to simulate community walking environments. Two researchers followed each participant, with one recording the video and another providing hand signals at the start and stop points (detailed below). Terrains included a path of large (16”) pavers, small (7”) pavers, mulch, gravel (upslope and downslope at a 9% grade), and sand (distances provided in Fig 1). All pathways were measured with a tape measure, and flags were placed as markers to be visible on video recording. Similarly to the 10MWT, flags were placed after the start and before then end of each walkway to account for any acceleration, deceleration, or transitions between terrains.

Participants performed one clockwise and one counterclockwise loop. Passes were averaged to account for curvature of the path for all conditions, although the upslope and downslope gravel conditions were included only from the counterclockwise and clockwise passes, respectively. If a participant stopped in the middle of a terrain, only data from the other direction were used for that terrain. This occurred for three participants (two in the large paver condition and one in the mulch condition). Participants used the same assistive device, prosthetic, and/or orthotic that was used on the indoor 10MWT.

Prior to testing, a researcher provided the following standardized verbal instructions: “I would like for you to walk around the course at a pace that is comfortable and safe for you.” Participants received verbal and visual cues while on the course directing them to the next terrain. During a pass, participants were allowed to take a standing rest between terrains if needed. Between the clockwise and counterclockwise passes, participants were allowed to take a standing, leaning, or sitting rest breaks for up to 5 minutes, if requested. Even though terrain distances (as short as 4.2 meters in the sand conditions) varied from the 10 meters used for the 10MWT, previous literature indicates that the 4-meter walk test and 10MWT demonstrate excellent agreement for single-trials of gait speed. Although those authors cautioned against using the 4-meter walk test and 10MWT interchangeably in a clinical setting, this was because of concern that the variability in measurement could mask small but clinically significant changes in gait speed [28]. We deemed that comparison of the 10MWT to shorter distances was acceptable in our case given that this would make differences less likely in our sample. Therefore, we anticipated that the presence of differences between conditions would likely constitute a true difference.

### Data processing

Video recordings were processed by two investigators who noted start and finish times for each terrain to the nearest tenth of a second and counted the number of steps taken, reaching consensus. A third investigator then independently confirmed data processing. Video estimation of spatiotemporal aspects of gait has been shown to be valid [29] and highly reliable [30]. Data were processed for the outdoor challenge course as a continuous loop, but videos were paused after each terrain to allow for accurate assessment of spatiotemporal data. For each terrain, time began and ended when the participant’s torso was in line with the marker. When counting the number of steps, the first step was counted when the foot was more than halfway past the marker (heel or completely past). Steps were then counted to the last step prior to the final marker. If the last step was in line with the marker, it was only counted if the foot was less than halfway past the marker. When fully visible in the video, the markers laid out indoors and on the outdoor course were used to establish start and finish points. Otherwise, the investigator’s hand signal was used for start/stop times. These data were then used to calculate gait speed (path distance/elapsed time), gait cadence (number of steps/elapsed time), and average step length (path distance/number of steps) and WR (step length in cm divided by cadence [cm/steps/min]).

### Statistical analysis

All statistical analyses were performed using R [31]. The R code used for statistical analysis is available at https://github.com/RegainLabUNC/SpatioTemporal-Changes-Across-Terrains. Initial versions of R code used for statistical analysis were created with the help of artificial intelligence (Open AI, ChatGPT [GPT-4o]). However, all code was assessed for completeness and accuracy by the investigators, and all data were analyzed and interpreted by researchers without the use of AI.

We performed repeated-measures ANOVA using the rstatix package [32] to analyze differences in gait speed, cadence, average step length and WR between each terrain and the baseline 10MWT condition. Sphericity was assessed using Mauchly’s test of Sphericity, and a Greenhouse-Geisser correction was used when sphericity was violated. Shapiro-Wilks test was used to determine normality for each condition. Normality was confirmed for all data except cadence in the large paver condition. However, given that ANOVA is robust to skew with sample sizes greater than 30, all statistical assumptions were met. Effect sizes were based on Cohen’s interpretation of a partial eta squared (η_p_^2^) and Cohen’s *d* [33]. Post-hoc pairwise comparisons were performed with Bonferroni adjustments, comparing each terrain to the 10MWT.

To determine how the changes in cadence and step length affected gait speed changes, these variables were placed into a stepwise, multiple linear regression for each condition. Model fit was assessed using R-squared (R^2^) and weighting of parameters in each model was assessed using standardized coefficients. For a given terrain, we created a standardized regression model that included change scores for step length and cadence as the predictor variables and change in gait speed from the 10MWT as the outcome variable. Multicollinearity was assessed using variance inflation factors of step length and cadence using the car package in R [34]. For each model, we assessed the assumptions of normality, homoskedasticity, and linearity using quantile-quantile plots and residual vs fitted plots for each model. We noted heteroskedasticity, so t-tests of regression coefficients used heteroskedasticity consistent standard errors (HSC SE). Likewise, we noted that some relationships appeared non-linear. However, the addition of a polynomial term did not reach statistical significance using HSC SEs. Therefore, the linear interpretation of each model demonstrated the best fit. Adjusted alpha level for all statistical tests was set at 0.05.

## Results

### Gait speed, cadence, average step length, and walk ratio

**Fig 2:**
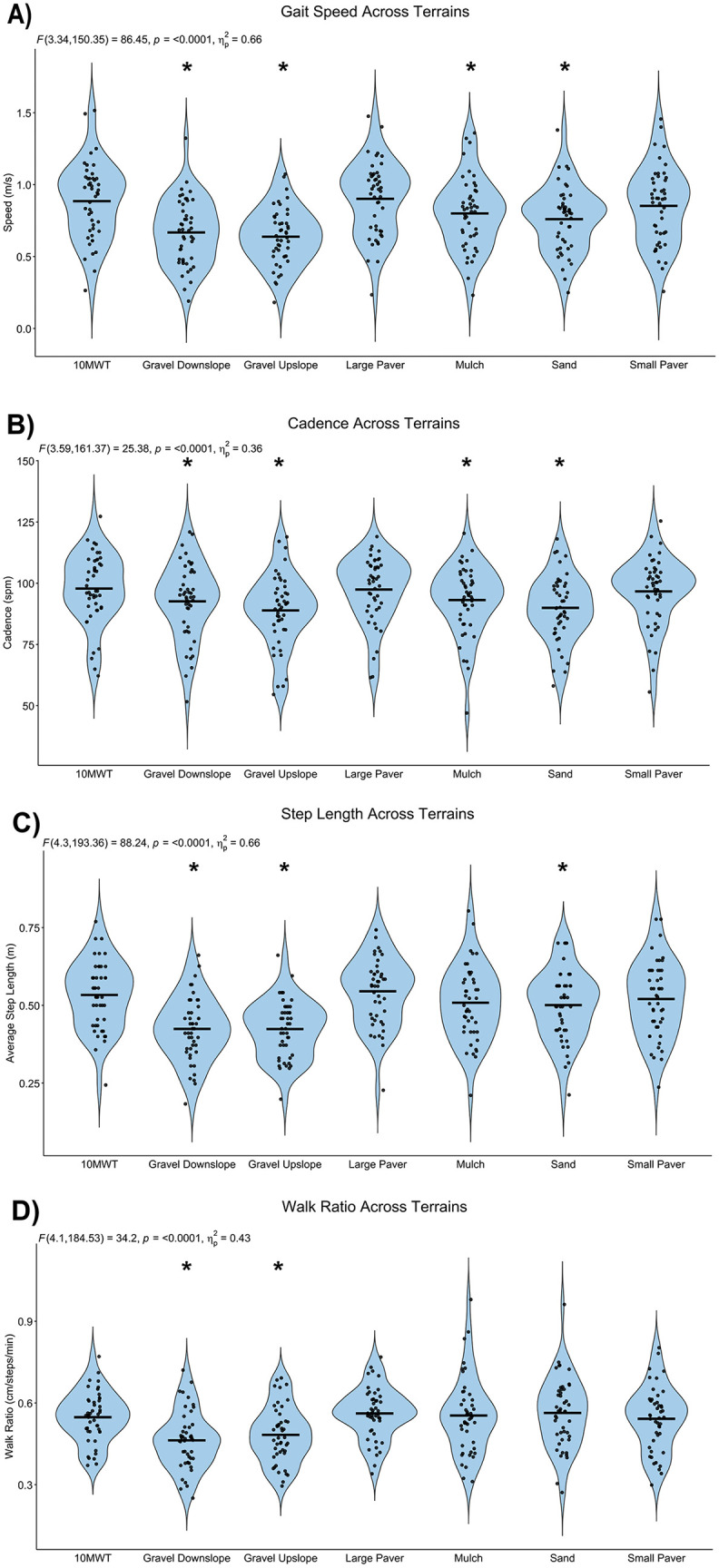
Average Gait Speed (A), Cadence (B), Step Length (C), and Walk Ratio (D) across Terrains; Violin plots with individual participant data of A) gait speed, B) cadence, C) average step length, and D) walk ratio across terrains. *indicates statistically significant compared to 10MWT condition.

All gait variables are presented in Table 2. During the baseline 10MWT, participants walked at an average (standard deviation) gait speed of 0.89 (0.26) meters per second with an average cadence of 97.81 (14.34) steps per minute, and an average step length of 0.53 (0.11) meters. Overall, we observed a significant main effect for terrain on gait speed (F(3.34, 150.35)= 86.45, p < .001, η_p_^2^ = 0.66), cadence (F(3.59, 161.37)= 25.38, p < .001, η_p_^2 ^= 0.36), average step length (F(4.3, 193.36)= 88.24, p < .001, η_p_^2 ^= 0.66), and WR (F(4.1, 184.53)= 34.20, p < .001, η_p_^2^ = .43). We observed slower gait speed during the sand (p < .001, *d* = 0.96), gravel downslope (p < .001, *d* = 1.43), gravel upslope (p < .001, *d* = 1.60), and mulch (p= < .001, *d* = 0.67) conditions compared to the 10MWT (Fig 2A). In contrast, no difference in gait speed was noted between the 10MWT and the large paver (p = 1.00, *d* = 0.17) and small paver (p = 1.00, *d* = 0.26) conditions. Similarly, no differences were seen in cadence between the 10MWT and the large paver (p = 1.00, *d* = 0.07) and small paver (p = 1.00, *d* = 0.13) conditions but, participants showed a slower cadence in the sand (p < .001, *d* = 0.85), gravel upslope (p < .001, *d* = 0.95), gravel downslope (p = .03, *d* = 0.51), and mulch (p = .003, *d* = 0.61) conditions (Fig 2B). We noted decreased step lengths when walking in the sand (p = .01, *d* = 0.54), gravel downslope (p < .001, *d* = 1.80), and gravel upslope (p < .001, *d* = 1.69) conditions compared to the 10MWT. No differences were noted in the large paver (p = 1.00, *d* = 0.30), small paver (p = 1.00, *d* = 0.23), or mulch (p = .31, *d* = 0.37) conditions. Finally, we noted significant differences in the WR in the gravel downslope (p < .001, *d* = 1.19) and gravel upslope (p < .001, *d* = 0.90) conditions. However, no significant differences in WR were noted in the large paver (p = 1.00, *d* = 0.28), small paver (p = 1.00, *d = *0.08), sand (p = 1.00, *d* = 0.19), and mulch (p = 1.00, *d = *0.07) conditions.

**Table 2:** Spatiotemporal Gait Parameters Across Terrains.

Terrain Distance in m	Number of Participants	Gait Speed in m/sec, Mean (SD)	Gait Cadence in steps/min, Mean (SD)	Step Length in m, Mean (SD)	Walk Ratio in cm/steps/min, Mean (SD)
**10MWT: Smooth, Level, Indoor 10 m**	46	0.89 (0.26)	97.81 (14.34)	0.53 (0.11)	0.55 (0.09)
**Large Paver,** **21 m**	46	0.90 (0.26)	97.40 (13.60)	0.55 (0.11)	0.56 (0.09)
**Sand** **4.2 m**	46	0.76 (0.23)	89.92 (13.55)	0.50 (0.11)	0.56 (0.13)
**Gravel, Downslope** **11.9 m**	46	0.67 (0.23)	92.58 (15.41)	0.42 (0.10)	0.46 (0.11)
**Gravel, Upslope** **11.9 m**	46	0.64 (0.20)	88.90 (14.83)	0.42 (0.09)	0.48 (0.10)
**Mulch** **6 m**	46	0.80 (0.25)	93.11 (14.09)	0.51 (0.12)	0.55 (0.14)
**Small Paver** **5.8 m**	46	0.85 (0.27)	96.65 (13.92)	0.52 (0.12)	0.54 (0.12)

Summary of Gait Spatiotemporal measures; cm (centimeters), m (meters), m/sec (meters per second), min (minute)

### Spatiotemporal mechanism for gait speed change

Multiple regression revealed that the change in gait speed from the 10MWT was related to both the change in step length and change in cadence for all conditions (p < .001). Regression coefficients, standardized regression coefficients, and R^2^ values are reported in Table 3. As expected, the combined change in step length and change in cadence explained between 95 and 99% of the variance of the change in gait speed across the different terrains. Standardized coefficients for change in step length (β_Step Length_) and change in cadence (β_Cadence_) were statistically significant (p < .001) across terrains. Specifically, we observed the following standardized coefficients: large paver (β_Step Length_ = 0.70, β_Cadence_ = 0.62, R^2^ = 0.98) small paver (β_Step Length_ = 0.74, β_Cadence_ = 0.60, R^2^ = 0.99), sand (β_Step Length_ = 0.72, β_Cadence_ = 0.63, R^2^ = 0.96), gravel downslope (β_Step Length_ = 0.68, β_Cadence_ = 0.54, R^2^ = 0.95), gravel upslope (β_Step Length_ = 0.76, β_Cadence_ = 0.48, R^2^ = 0.95), and mulch (β_Step Length_ = 0.82, β_Cadence_ = 0.55, R^2^ = 0.98).

**Table 3:** Results of Multiple Linear Regression.

Walking Condition	Unstandardized Coefficients b_1_: Step length change	Unstandardized Coefficients b_2_: Cadence change	Standardized Coefficients β_StepLength_: Step length change	Standardized Coefficients β_Cadence_: Cadence change	R^2^ Total Variance Explained
**Large Paver**	1.59	0.01	0.70	0.62	0.98
**Sand**	1.57	0.01	0.72	0.63	0.96
**Gravel, Downslope**	1.70	0.01	0.68	0.54	0.95
**Gravel, Upslope**	1.80	0.01	0.76	0.48	0.95
**Mulch**	1.59	0.01	0.82	0.55	0.98
**Small Paver**	1.67	0.01	0.74	0.60	0.99

Unstandardized and standardized regression coefficients for the contributions of change in step length and change in cadence on predicted change in gait speed across terrains. All regression coefficients and R-squared values were statistically significant (p < .001)

## Discussion

### Gait spatiotemporal changes across terrains

Participants reduced their gait speed in the sand, mulch, and both sloped gravel conditions compared to an indoor 10WMT. These gait speed changes coincided with reduced cadence in the sloped gravel, mulch, and sand conditions and with decreased step lengths in the sloped gravel and sand conditions. Thus, our hypothesis that participants’ gait speed, obtained initially using the 10MWT in the clinic, would decrease when navigating various outdoor terrains was partially supported. Notably, average gait speed changes were at, or comparable to, the minimal detectable change for gait speed for most diagnoses included in the study, indicating that the observed gait speed changes may be both statistically and clinically significant [35–42]. Overall, these data suggest that more compliant and sloped surfaces (i.e., mulch, sand, and gravel) have a greater influence on spatiotemporal measures of gait than outdoor surfaces that were firm and level (i.e., large and small pavers). These findings indicate that the 10MWT assessed in a clinical setting best reflects walking performance on outdoor surfaces that are sufficiently similar to the testing environment (i.e., firm and level surfaces). However, it may not fully reflect gait on more challenging outdoor terrains that are less like the indoor level conditions used to standardize the 10MWT.

### Changes in step length and cadence

As expected, changes in step length and cadence explained most of the variance in changes in gait speed compared to the 10MWT (95–99% across conditions). We also noted that the WR significantly decreased in both sloped gravel conditions but remained unchanged over all other terrains. Bogan et al. reported that walk ratios for unimpaired community-dwelling older adults do not change when walking on uneven terrains [20]. Our findings of decreased gait speed in the sand and mulch conditions without changes in the WR corroborate these findings across a broader range of diagnoses affecting gait. Yet, the combination of slope and gravel appeared to alter the strategy used by our participants to modulate gait speed.

Slopes are well-known to affect spatiotemporal aspects of gait, with previous evidence of decreased speed, step length, and cadence when walking on an upslope [43–45]. The observed decrease in cadence in the gravel downslope condition conflicts with previous literature in unimpaired individuals which generally shows an increase in cadence with downslope walking (presumably to maintain speed) [43,44]. This may be explained by the concomitant decrease in walk ratio, indicating that the combination of slope and uneven terrain may alter the control strategy used to adapt to very challenging terrains. We can speculate that individuals with mobility deficits select this strategy to demonstrate a more cautious approach to navigating down sloping terrains, proportionally decreasing step length more than cadence.

Standardized regression coefficients revealed that step length changes appeared to be more heavily weighted than cadence changes to alter gait speed, despite varying degrees of difference. In some conditions (e.g., large paver, small paver), step length and cadence changes demonstrated similar contributions to gait speed change while step length change demonstrated a much larger contribution in others (e.g., the upsloping gravel conditions). To manipulate gait speed, individuals can change both step length and cadence while walking [46]. For example, unimpaired individuals can maintain gait speed by decreasing step lengths and increasing cadence on a treadmill, which may improve the margin of stability [47]. Our data suggest that despite the ability to change gait speed with both step length and cadence, step length changes appear to contribute to larger changes in gait speed compared to cadence. However, both variables changed proportionally in conditions where WR did not change.

In the sloped gravel conditions, we can speculate that the decrease in WR may reflect a strategy intended to proportionally decrease step length relative to cadence to increase gait stability. This is further supported by the greater weighting of step length in modulating gait speed change in these conditions. Changing both step length and cadence, however, can influence walking stability [47–49]. It is possible that walking on mulch may not have perturbed balance enough to require a modulation in step length, given that walk ratio remained unchanged from the 10MWT but still led to a decrease in cadence. Except for the sloped gravel conditions, our findings suggest that individuals with mobility concerns do not alter their WR when walking over level challenging terrains like mulch and sand.

Overall, individuals with mobility deficits walked differently on outdoor terrains that included slopes, gravel, mulch, and sand compared to the indoor 10MWT. Though our findings do not examine individuals in their own community environments, these data indicate that gait performance may differ on uneven, outdoor terrains compared to standardized indoor clinical measures of gait performance. Likewise, others have shown that gait variability [50–53], joint work [52], muscle activity [52,54], and whole-body angular momentum [50,53] also change when walking on uneven terrains. Clearly, walking performance on unlevel surfaces may not be fully reflected by walking under the conditions of clinical measures like the 10MWT. Despite these changes, the underlying mechanism by which gait speed is controlled (walk ratio) remained relatively unchanged except when participants walked over sloped gravel.

### Strengths and limitations

Interpretation of this study should consider certain limitations with our design. First, sample size was based on a convenience sample rather than a priori power calculation. Nevertheless, we recruited a relatively robust sample size and report effect sizes to aid in the interpretation of our results. Second, the effect of supervision from the physical therapist and investigators during sessions could have unintentionally influenced participants’ speed on the course [55], either through the perception of pacing or increased confidence due to the therapist’s presence. Given that some participants required contact guarding or close supervision for safety, this was an unavoidable limitation. Similarly, assessing outdoor walking during a physical therapy session may have led participants to walk slower towards the end of the session. The fact that the large and small paver conditions (at the beginning and end of the course) did not differ from the 10MWT, though, indicates that there was likely no ordering effect. Similarly, we averaged passes in each direction to minimize the effects of fatigue and curvature of the path. We also used video-based estimates of gait speed, step length, and cadence which may overestimate spatiotemporal aspects of gait compared to other instrumented measures [56,57]. However, previous literature has demonstrated that this method demonstrates good inter- and intra-rater reliability when estimating spatiotemporal aspects of gait [30]. As our method was kept consistent between participants and terrains, our data should accurately reflect the magnitude of change. Additionally, although other methods such as the use of markerless motion capture or inertial measurement units may offer accurate results, our method is more consistent with clinical practice at this time. Rehabilitation care has historically relied on very basic measurement tools (i.e., stopwatches and visual estimation) to measure gait quantity and quality, and this remains the norm in standard physical therapy practice today. Therefore, these data are directly interpretable for physical therapists and other rehabilitation practitioners across a broad range of populations. Unlike other studies which have focused on single clinical populations [18,54,58] or unimpaired adults [51,59], these results are generalizable to the treatment of a broader range of mobility deficits. To our knowledge, no other study has examined changes in gait on real-world surfaces compared to the 10MWT across a diverse sample of diagnoses, reflecting conventional practice.

## Conclusions

The findings of this study indicate that a standardized measure of walking capacity like the 10MWT may not reflect an individual’s chosen gait speed or underlying spatiotemporal characteristics, while walking on challenging terrains commonly encountered in the community such as gravel, mulch, sand, and slopes. The reduction in gait speed appeared to result from proportional changes in both step length and cadence while on level surfaces but may reflect a more cautious gait pattern on sloped surfaces. Rehabilitation specialists should take this into account when training and preparing individuals for discharge.
